# Penicillin Allergy Skin Testing in the Inpatient Setting

**DOI:** 10.3390/pharmacy7030120

**Published:** 2019-08-27

**Authors:** Julie Ann Justo, Wesley D. Kufel, Lisa Avery, P. Brandon Bookstaver

**Affiliations:** 1 Department of Clinical Pharmacy and Outcomes Sciences, University of South Carolina College of Pharmacy, Columbia, SC 29208, USA; 2 Department of Pharmacy, Prisma Health Richland Hospital, Columbia, SC 29203, USA; 3 Department of Pharmacy Practice, Binghamton University School of Pharmacy and Pharmaceutical Sciences, Binghamton, NY 13902, USA; 4 Department of Medicine, State University of New York Upstate Medical University, Syracuse, NY 13210, USA; 5 Department of Pharmacy, State University of New York Upstate University Hospital, Syracuse, NY 13210, USA; 6 Department of Pharmacy Practice, Wegmans School of Pharmacy, St. John Fisher College, Rochester, NY 14618, USA; 7 Department of Pharmacy, St. Josephs Health, Syracuse, NY 13203, USA

**Keywords:** allergy, penicillin, skin testing, hospitalization, antimicrobial stewardship, beta-lactam

## Abstract

The consequences of a documented penicillin allergy in the medical record are especially troublesome in acutely ill, hospitalized patients. A penicillin allergy label may lead to alternative or second line therapies resulting in adverse drug events, negative clinical outcomes and increased costs. Reconciling penicillin allergies is a necessity to facilitate early, optimal therapy and is a shared responsibility among the healthcare team. Penicillin skin testing (PST) has been utilized successfully in hospitalized patients to de-label erroneous penicillin allergies and optimize antibiotic therapy. This targeted review aims to discuss the practical development and implementation of PST in the inpatient setting. This includes a needs assessment checklist with common considerations allowing for customization to one’s institution based on available personnel, time, and technological resources.

## 1. Introduction

An estimated 30 million Americans acknowledge a penicillin allergy in their medical record, while more than 90% of these are considered to be falsely labeled [1]. The potential impact of a penicillin allergy label cannot be underestimated, especially in hospitalized patients. In 2014, over 35 million inpatient stays were documented in United States (US) hospitals [2]. With septicemia and pneumonia ranking third and sixth among principal diagnoses for inpatient stays, a documented penicillin allergy may negatively impact many hospitalized patients [2]. Penicillin allergies have been associated with worse clinical outcomes, inflated healthcare costs and more frequent secondary infections with drug-resistant pathogens [3,4,5]. This should come as no surprise given the number of infections for which beta-lactams, including penicillin derivatives and narrow-spectrum cephalosporins, serve as the evidence-based, preferred treatment [6,7,8]. Patients with a documented penicillin allergy often receive alternative antibiotics (e.g., vancomycin, clindamycin, gentamicin, fluoroquinolones, macrolides), some with inferior effectiveness in indications such as surgical prophylaxis, and some with higher probability of adverse effects, such as *Clostridioides difficile* infection [3,4].

Given the large number of estimated penicillin allergies that are falsely labeled, the ability to reconcile the allergy and broaden available antibiotic options for hospitalized patients is key. Managing penicillin-allergic patients in the inpatient setting, an important function of antimicrobial stewardship programs (ASPs), may come in many forms depending on the patient and institution, but it should always begin with a comprehensive allergy reconciliation and patient assessment (Table 1). Penicillin skin testing (PST) is a favorable option that rules out the risk of a positive IgE-mediated reaction with a more than 95% negative predictive value [9,10,11]. Herein, we describe the practical development and implementation of PST in the inpatient setting. This includes a needs assessment checklist with common considerations allowing for customization to one’s institution based on available personnel, time, and technological resources. This review includes relevant references identified via PubMed using search terms including “skin tests”, “penicillins”, and “inpatients”; yet, does not represent a comprehensive review of all available literature. Detailed reviews of PST in the outpatient setting, as well as reviews of allergy and cross-reactivity with cephalosporins, carbapenems, and beta-lactamase inhibitors are described elsewhere [12,13,14,15].

## 2. Penicillin Allergy Skin Testing Procedure

PST has long been conducted in the outpatient setting, primarily by allergists [12]. Introduction into the inpatient setting was a more recent development and not until 2004 was there published evidence of pharmacist involvement [16]. The standard PST procedure is a multistep process utilizing both minor and major determinant antigens, which takes approximately 45–60 min to complete [9,11]. Individual determinants of penicillin allergy were evaluated beginning in the 1950s and 1960s [17,18]. Early on it was described that penicillins, including benzylpenicillin, break down to a protein derivative (i.e., penicilloyl), which is the major allergenic determinant for IgE-mediated reactions. Testing with a major determinant alone is insufficient for PST, as 36 to 68% of patients with a penicillin allergy history tested positive to the multivalent form of the major determinant, penicilloyl-polylysine [17]. Potentially life-threatening immediate reactions may occur in patients secondary to IgE response to minor determinants, necessitating their need for PST in combination [17]. Prior data also suggest that few patients may only test positive to amoxicillin, although less commonly in the US compared to Europe [17,19,20].

Before beginning PST at your institution, a needs assessment among interested stakeholders should be performed to answer several key questions and develop appropriate protocols (Table 2). The key elements that need to be determined include target patient populations, personnel involved to perform the tests, inclusion/exclusion criteria for patients, training and credentialing of personnel, informatics/IT infrastructure needed, and response to both a negative and positive test [9,21].

**Figure 1:**
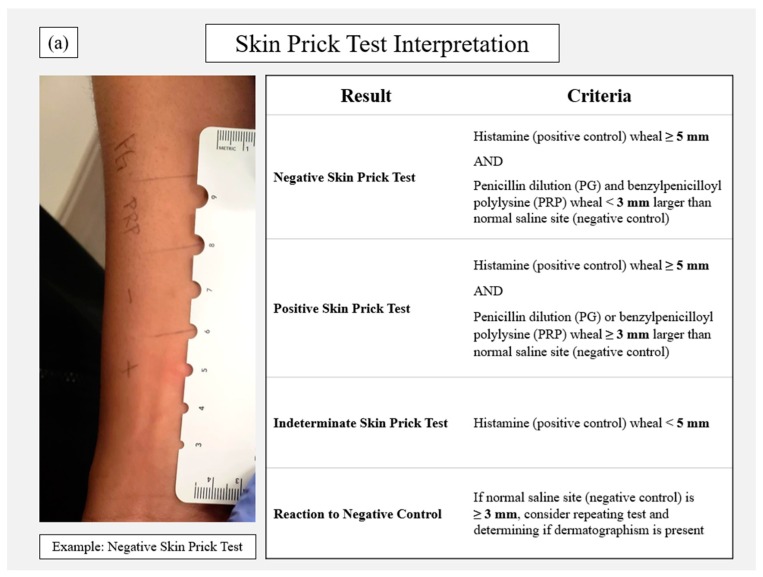

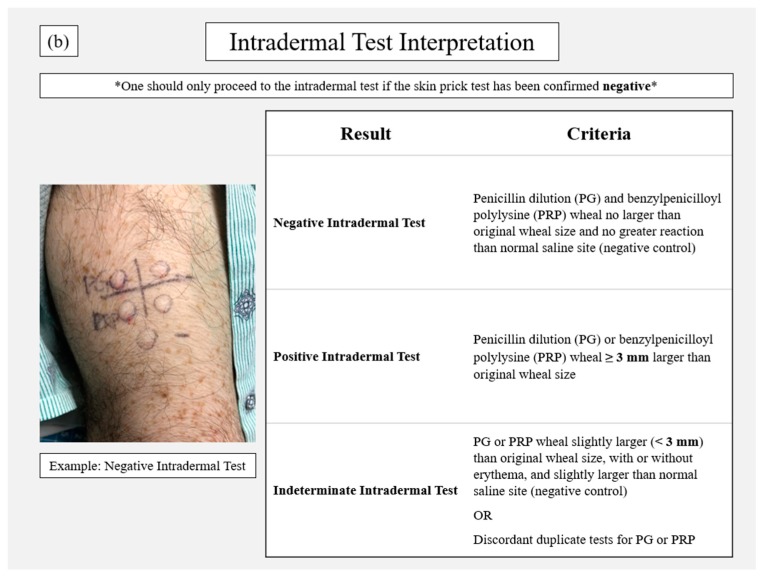
Penicillin skin testing procedure including (**a**) step 1 of the skin prick test and (**b**) step 2 as the intradermal test with associated interpretation thresholds for each.

Identifying the personnel involved in performing the test is an important component in the protocol development phase. Pharmacists, physicians and nurses have all been successfully used, with varying degrees of pharmacist involvement. Depending on the personnel involved, some institutions and states will have regulations that may prohibit or place restrictions on specific healthcare workers. Consulting with local regulatory boards is advised prior to initiation [9]. A national certificate program is newly available to train and certify interested personnel on allergy reconciliation and PST procedures [21]. The PST process is described in Figure 1 and available elsewhere in detail [11,22]. In brief, following comprehensive allergy reconciliation, the patient should be screened against institutional inclusion and exclusion criteria. Consent should also be obtained prior to performing the test as appropriate. Procure all supplies from the pharmacy (Table 3). The test can be performed at the bedside in a two-step modality: (1) a prick test, typically on the forearm, followed by (2) an intradermal test, often on the upper arm, if the prick test results are negative. The prick test involves four components: histamine (positive control); saline (negative control); benzylpenicilloyl polylysine (major determinant); and dilute penicillin (minor determinant) [11]. The forearm should be prepped with an alcohol swab and labeled to document where a single drop should be placed for each of the four components (Figure 1a). Using a skin prick device, a single turn should be made through each drop. A new device should be used for each component to avoid cross contamination. The patient should feel a small “sting”, often described as equivalent to a mild insect bite. Measurements should be taken after a 15–20-min waiting period. A negative prick test means the patient has a positive reaction to the histamine and a negative reaction to the other three components. Because histamine is used as the positive control, any histamine H1 receptor antagonists (e.g., diphenhydramine, hydroxyzine) should be avoided for at least 48 h prior to the test. Recent ingestion of antihistamines is often the reason for a non-reactive or indeterminate test (i.e., <5 mm reaction to the histamine). Many centers also suspend other medications with antihistaminic properties prior to the test when possible, such as tricyclic antidepressants (e.g., amitriptyline, nortriptyline) and atypical antipsychotics (e.g., aripiprazole, olanzapine, quetiapine) [23]. Patients receiving non-selective beta-blockers may also be excluded from some protocols or require consultation with an allergist prior to PST. The intradermal test should only be conducted following a negative reaction to the skin prick test [11].

The intradermal test utilizes the same components, excluding histamine. The skin should be prepped (alcohol swab) and labeled appropriately for each component (Figure 1b). Using the syringe and intradermal needle, a small bleb (~3 mm) should be made for each component: single bleb for normal saline (negative control) and duplicate blebs for dilute penicillin and benzylpenicilloyl polylysine. Following injection, each bleb should be encircled with a pen to allow for measurement of bleb extension to determine a positive or negative reaction (Figure 1b). In cases of a severe reaction to the test, on-call or at bedside rescue medications should be readily available with procedures in place if the need for rapid response occurs (Table 3). The chance of a systemic reaction occurring from PST is exceedingly rare, reported as 0.16% among nearly 20,000 patients exposed to both major and minor determinants [11,17]. Deviations from standard two-step protocols should be conducted by trained allergists.

**Figure 2:**
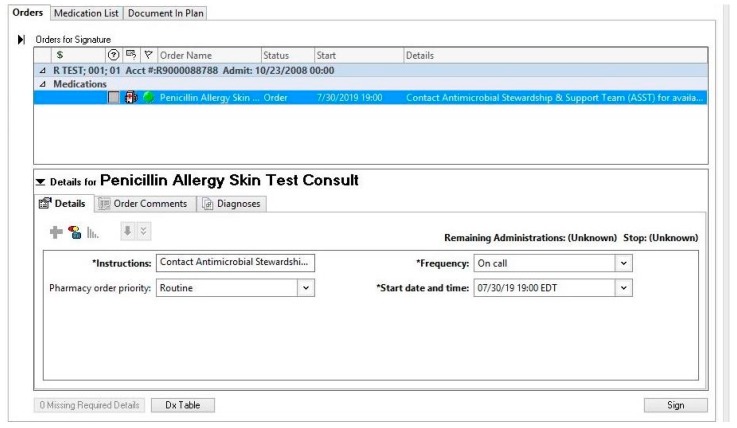
Penicillin allergy skin test consult order via computer physician order entry.

**Figure 3:**
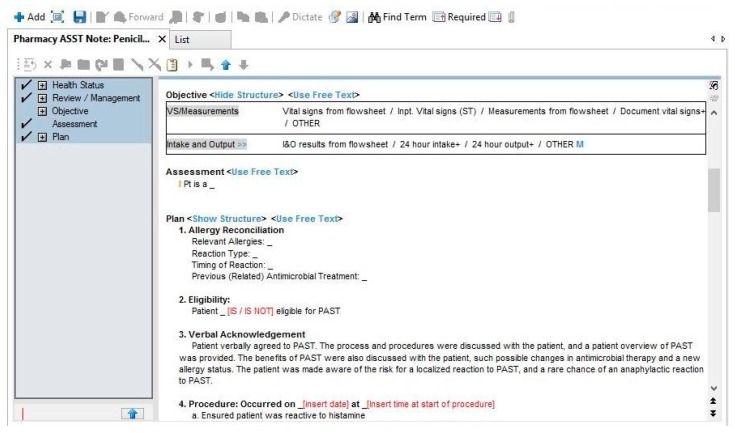
Sample portion of penicillin allergy skin test template note.

**Figure 4:**
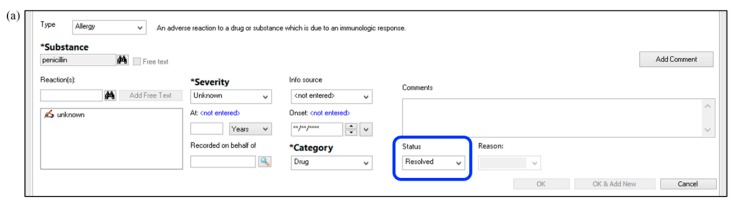

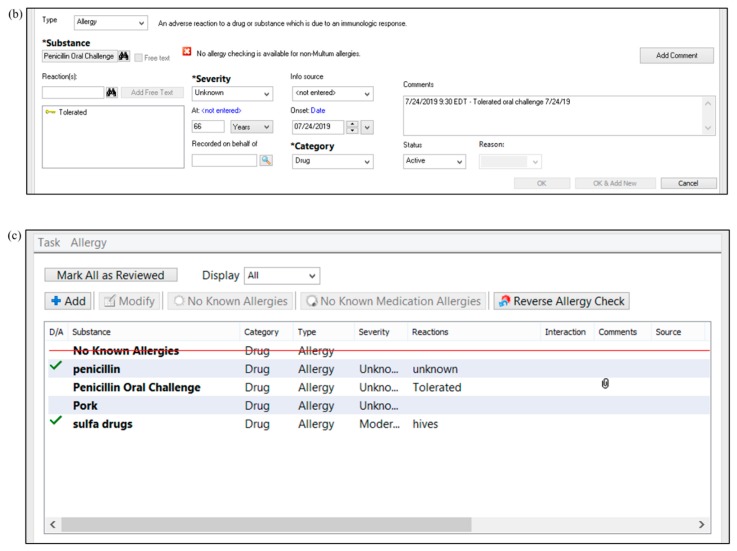
Process for penicillin allergy de-labeling in the electronic medical record by (**a**) changing the status of the current penicillin allergy to “resolved”, then (**b**) entering a placeholder for a tolerated penicillin oral challenge (or for a negative penicillin allergy skin test, as applicable). Panel (**c**) shows the display of “all” allergies for the patient, both active and inactive (e.g., resolved). The patient’s demographic bar in the electronic medical record is set to display only “active” allergies by default. In the “active” display, the penicillin oral challenge label would remain, but the penicillin allergy label would no longer be seen.

The time allowed before reading the results of each step of the PST is 15–20 min, during which time details on the patient’s outpatient pharmacy and specialty providers (e.g., dentists, specialists) may be collected if not already done. The medical record note may also be initiated to increase efficiency. A total test time of approximately 45 min is required for the final results [9,24]. Documentation of the results in the medical record, updating of the allergy record, providing patient education and notification of other providers is important. The current standard of care for many PST protocols is to include the administration of an oral challenge with amoxicillin [10,22,25]. The primary utility of an oral challenge following PST is to rule out a non-IgE-mediated reaction (e.g., urticaria) that is often delayed and self-limiting. This need is often mitigated if a switch to a penicillin-type antibiotic is indicated as therapy for the patient’s current infection. An oral challenge with amoxicillin also has the added benefit of ruling out patients who tolerate penicillin, yet may have amoxicillin-specific reactions. Though reported, this combination is exceedingly rare, especially in the US [20,26]. In cases where the patient is currently requiring antibiotic therapy, it is important to use the PST results to optimize antibiotics as soon as possible. If pharmacists or physicians who are not accustomed to documenting medication administration in the medication administration record (MAR) are performing the tests, there should be training and accessibility in place for barcoding or other documentation methods to be followed in accordance with institutional policy. This is not typically an issue for nurses who are accustomed to the MAR charting. Figure 2, Figure 3 and Figure 4 show several examples of PST protocol orderables and documentation of allergy reconciliation.

## 3. Customization by Institution

Most published PST services describe variations based on the resources and needs of the local institution. A similar process of customization is encouraged when completing the needs assessment checklist (Table 2) for development of a new PST service. As with most quality improvement initiatives, local institutions should consider three essential resources when developing a PST service: personnel, time, and technology. In addition, demand will be driven by the local patient population(s) at highest risk of negative consequences from spurious penicillin allergy labels.

### 3.1. Personnel

A variety of hospital personnel are capable of performing allergy interviews and PST in the inpatient setting. The local personnel identified to perform PST is often based on personnel goals, expertise, and availability. The goals and expertise of a PST service align naturally with those of allergy and immunology services; thus, allergists and allergy-trained nurses are typically incorporated when available. However, given the limited availability of allergists in many inpatient settings, numerous allergists support the expansion of PST services by other trained healthcare professionals, such as pharmacists, infectious diseases (ID) fellow physicians, nurses, emergency medicine physicians, or a combination of providers [9,10,13,27,28,29,30,31]. A growing number of successful PST services now function as part of antimicrobial stewardship programs [23,27,28]. These antimicrobial stewardship personnel aim to optimize antimicrobial use and possess the expertise to maneuver the unintended consequences of a specific patient’s penicillin allergy label. They are also experienced in collating and reporting outcome measures for the institution regarding the impact of allergy-related initiatives (e.g., antimicrobial use, antimicrobial resistance, time to appropriate therapy, incidence of *Clostridioides difficile* infection).

### 3.2. Time

Personnel also need the time to assess, perform, and manage PST consults. Time estimates have been provided for select aspects of a PST service, e.g., conducting an allergy interview (14 min), performing both the prick test and intradermal tests (45 min), and performing a drug challenge (1–5 h) [10,24,25,29,30]. Thus, provider time with the patient will typically average around 2 h, assuming either an oral challenge or the first dose of an intravenous beta-lactam is monitored at the bedside for 60 min. However, there is a paucity of data outlining time commitments for a complete inpatient PST consult as described herein. Time must be added for reviewing and corroborating health records as part of the allergy reconciliation, preparing PST components in the pharmacy, providing patient/provider/pharmacy education on PST results, updating both internal and external medical records, billing, etc.

In a survey of 50 ID fellowship program directors in the US, inadequate personnel and time were listed as two of the main barriers to implementing inpatient ID fellow-led PST services at their institution, highlighting the importance of time as part of a baseline needs assessment for a local PST service [31]. In addition, a large academic medical center in the Northeastern US described being able to perform PST for only 24% (43/179) of eligible patients during their hospitalization. Lack of time and logistical challenges, e.g., patient being discharged or transferred prior to coordination of PST, were the main reasons the remaining 76% of patients did not have a PST performed [29].

One strategy to maximize time is to leverage general staff (e.g., other nurses, pharmacists, physicians, trainees) to complete some of these tasks before and/or after the PST and drug challenge. These additional personnel must also be accounted for in any baseline needs assessment. For example, 4th-year pharmacy students scheduled in the hospital for an experiential education rotation can interview patients regarding their penicillin allergy using a standardized form, obtain outpatient pharmacy and primary care provider information, and contact those entities with the updated allergy information following the results of the patient’s PST. This model can be adapted to include any combination of available healthcare professionals and/or trainees capable of performing allergy assessment interviews and documentation as part of patient care.

Another aspect that may liberate personnel time is to demonstrate cost-savings for the institution and/or bill for PST services rendered. Physicians and nurses are usually able to bill for performing the PST service; yet, pharmacist-performed PST typically only bills for the cost of supplies to perform the test. Fortunately, there are some published examples demonstrating cost-effectiveness of PST services, which should allow even a pharmacist-led service to justify the time spent on such a program [27,32,33]. Jones et al. described an average antimicrobial acquisition cost-saving of $353.03 per patient among all who received a PST at their local community hospital, even after considering PST drug supply costs of $140 per test [27]. King et al. described a similar antimicrobial cost-savings of $297 per patient who was tested and switched to a beta-lactam antimicrobial [32]. A more detailed cost-effectiveness analysis by Mattingly et al. among patients with methicillin-susceptible *Staphylococcus aureus* (MSSA) bacteremia demonstrated a PST service was associated with cost-savings of $660 per patient plus an additional 0.07 quality-adjusted life year (QALY) per patient [33]. They estimated that any PST cost (including labor) of less than $959.98 per patient would represent cost savings among this particular patient population [33]. Further discussion regarding targeting PST in select patient populations is provided in Section 3.4.

### 3.3. Technology

Technology is a third key resource that can help expand the breadth and scope of an inpatient PST service. The first consideration is whether the local institution’s medical record is electronic, non-electronic (i.e., paper), or a hybrid. Many of the published orders, templates, allergy sections, and other PST materials for electronic medical records (EMRs) can be transformed into paper versions by a local institution, if needed. However, most EMRs will have a great capacity for information sharing, alerting systems, surveillance, documentation, data collection, and other related functions that can be leveraged for PST implementation. For instance, a health system with five campuses developed a pop-up alert, or “Best Practice Advisory (BPA)”, which would appear anytime a clinician attempted to order an antibiotic for a patient with a penicillin allergy. The alert would then direct the clinician to a computerized guideline (accessible via computer or mobile app), which would usher the clinician through the correct decision-making pathway and provide final recommendations on the management of a wide variety of beta-lactam allergies [29]. This would include a robust array of options such as direct full challenges or graded challenges among low risk patients, skin testing via allergist consultation among moderate risk patients, and avoidance of the targeted agent/class or desensitization via allergist consultation for high-risk patients. They also described customization of the guideline recommendations by campus, as not all campuses had the same capacity to perform PST. Finally, they developed a monthly electronic dashboard that reports outcomes such as BPA firing, webpage usage, and guideline completion. [29].

As another example, the authors of this manuscript leveraged the pharmacy consultation system within their local EMR, Cerner^®^, to allow providers to request a “Penicillin Allergy Skin Test Consult” for any inpatient with an indication for antibiotic therapy [28]. This was in addition to ongoing identification of PST-eligible patients by antimicrobial stewardship pharmacists as part of their daily prospective audit and feedback for other antimicrobial stewardship initiatives. An example of the electronic consult order is shown in Figure 2. This order included the days and times PST was available, i.e., business days from 7:00 a.m. to 4:30 p.m., for provider reference. Upon order verification by a general pharmacist, the consult order would generate a pop-up alert instructing the pharmacist to page the local antimicrobial stewardship team to notify them of the case. This health system also utilized the EMR to develop a template PST consult note for documentation (Figure 3) and a procedure for updating allergy documentation with PST and/or oral challenge results—thereby minimizing the risk of relabeling of penicillin allergies in the EMR (Figure 4). With these documentation methods, 0% (0/30) of patients with a negative PST were subsequently relabeled as penicillin allergic, despite 90% (27) of patients having ≥ 1 subsequent encounter with the local health system [28].

**Figure 5:**
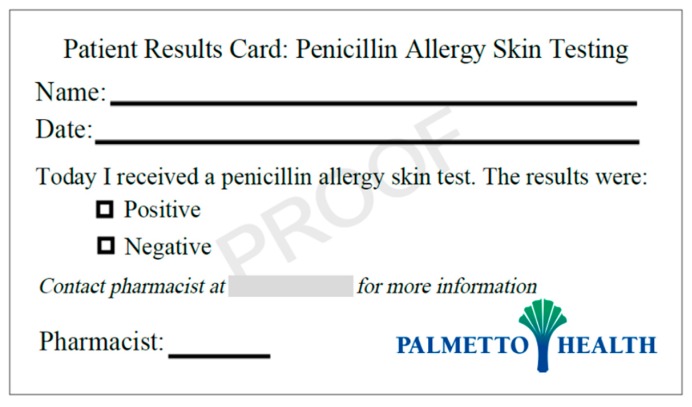
Sample patient education card following penicillin allergy skin test.

While technology is key for much of the PST process, low-tech options appear to work very well for education of patients, providers, and pharmacies regarding PST results. Numerous PST services have provided a small business card to patients for a quick reference regarding their PST results in the future [18,19]. A sample of such a card is shown in Figure 5. In addition, phone calls, faxes, and other routine communications to the patient’s outpatient pharmacy and external physician offices are key to ensuring the impact and benefit of PST efforts is maintained moving forward.

### 3.4. Patient Population

When developing a PST service, it is also important to customize the institutional protocol based on local demand, i.e., the estimated volume and type of patients anticipated to need PST. Given that 10–15% of hospitalized patients are estimated to have a penicillin allergy and that 1 in 2 hospitalized patients will receive an antibiotic during their hospital stay, there is a potential for a large volume of inpatients being eligible for PST. By having already outlined the available resources of personnel, time, and technology, a local service can likely anticipate the number of evaluations for PST (and actual tests) that can feasibly be performed in a given week. This capacity should inform which patient populations the PST service should target and what inclusion and exclusion criteria should be outlined for the PST protocol. Centers should consider developing a priority assessment tool to help stratify patients who would receive the greatest immediate benefit from PST based on available and allocated resources. Centers with a large PST capacity may allow evaluation for PST on all inpatients, while one with moderate capacity may only evaluate inpatients needing current antibiotic therapy, and another yet with limited capacity may only evaluate inpatients with serious infections where narrow-spectrum beta-lactams are considered drugs of choice (e.g., MSSA or enterococcal bacteremia).

Target patient populations can also be identified by chronic conditions at greater risk for adverse consequences of a spurious penicillin allergy, e.g., immunocompromised or critically ill patients, and/or by location of the patient, e.g., emergency department or preoperative screening. Each unique mix of target populations will lend its own preference and challenges to PST implementation. For instance, the PST service may employ drug challenges following PST more frequently in general inpatients on medical wards as the supervision needed is more readily available; yet, it may prefer PST alone for preoperative screening patients who are hospitalized for relatively short periods of time.

This leads to other pragmatic considerations regarding the flow of patients in the inpatient setting. If a PST service is alerted to a PST consult on a given day, the test is typically scheduled within the next 12 to 24 h, which often falls on the subsequent business day [23]. Other delays in the patient’s schedule may arise from the patient receiving drugs with antihistamine properties (e.g., diphenhydramine, tricyclic antidepressants) on the day of initial PST evaluation. Such exposure typically requires the medication(s) be held for 2–5 days before attempting PST in order to minimize the risk of the positive histamine control in the prick test failing to generate the required reaction [23]. These are just some of the circumstances that can delay scheduling of inpatient PST and increase the chance the patient is ready for discharge or transfer before the PST can be performed. Developing an alternative mechanism for outpatient PST (e.g., referral to an outpatient allergist) for such cases is recommended.

## 4. Conclusions

Given current efforts to optimize antimicrobial use and combat the emergence of antimicrobial resistance, there is a current call to action regarding reassessment of penicillin allergy labels among hospitalized patients. Fortunately, there are a growing variety of inpatient PST models to learn from [9,16,23,27,28,29,31]. Collectively, they demonstrate significantly improved clinical outcomes including increased use of preferred beta-lactam therapy for infection management, decreased healthcare costs, and increased quality-adjusted life years for patients. In order to ensure successful PST protocol development and implementation in a new inpatient setting, a needs assessment should be performed to identify available local resources, target patient populations, and other unique characteristics that will inform successful PST protocol development.

## Figures and Tables

**Table 1:** Key strategies for the management of penicillin-allergic patients in an inpatient setting ^a^.

**Allergy Reconciliation ^b^**
Comprehensive patient and/or family interviews regarding drug allergy
Medical record assessment and corroboration with external pharmacy and other records
Update of allergy documentation in the medical record
**Management**
Penicillin skin test (PST)
Graded challenge
Desensitization
Avoidance of target antibiotic and/or class, and administration of alternative antibiotic therapy

^a^ A single strategy is often used in combination with others in an individual patient case, e.g., allergy reconciliation, PST, and graded challenge. ^b^ Should be performed on all patients with a documented penicillin allergy.

**Table 2:** Needs assessment checklist for local penicillin skin testing (PST) development.

**Baseline Assessment**	
**Identify and assemble key stakeholders** with interest and expertise in allergy management and antimicrobial stewardship, such as: Allergists, infectious diseases physicians, and/or other physicians Infectious diseases pharmacists and/or other pharmacists Nurses and/or nurse practitioners Information Technology (IT) specialists/Clinical Informaticists	☐
**Identify personnel** who will execute PST and assess institutional and state regulations to determine if personnel are eligible (e.g., pharmacists, nurses, physicians)	☐
**Evaluate technology** available to aid in PST service development (e.g., paper vs. electronic medical records, alerting system, pop-up alert capacity, documentation options)	☐
**Determine time** when PST can be offered (e.g., Monday–Friday, 8 a.m. to 5 p.m.)	☐
**Determine target patient populations**, such as those currently receiving antibiotics, or with specified infectious diseases	☐
**Identify inclusion and exclusion criteria** for patients and projected minimum and maximum volumes for the service	☐
**Determine level of patient consent** required for PST	☐
**Implementation**	
**Determine training and credentialing process** of testing personnel	☐
**Determine alerting system** to notify personnel of a potential PST (e.g., consult, page)	☐
**Liaise with pharmacy staff** to outline PST supply procurement, storage, preparation, and dispensing	☐
**Collaborate with IT/clinical informatics** to ensure electronic medical record builds for appropriate PST ordering, alerting, etc.	☐
**Outline documentation methods** in the medical record, including updating of allergies following PST results *Recommendation:* Develop a PST note template for insertion into the medical record and a standard procedure for de-labeling (e.g., placeholder for a negative skin test and/or challenge)	☐
**Outline education** for patients, providers, and pharmacies (both internal and external) following PST results *Recommendation:* Develop materials for completion after PST interpretation: (1) a patient education card template (e.g., a small business card), (2) a patient questionnaire to collect external provider and pharmacy information	☐
**Determine institutional costs** per PST and identify processes for billing, if possible	☐
Identify appropriate approval pathways for the PST protocol within the local institution	☐
**Evaluation & Reporting**	
**Determine a process for data collection**, evaluation, and dissemination of PST service outcomes to: Justify sustainability of PST protocol Examine opportunities for improvement and expansion, as needed	☐

**Table 3:** Suggested components of a penicillin allergy skin test kit.

Component	Dose/Notation	Quantity ^a^	Comments
**Drug Product**			
Histamine base 1 mg/mL (Histamine phosphate 2.75 mg/mL) (positive control)	0.1 mL	1	For prick test
Sodium chloride 0.9% (negative control)	0.15 mL	2	For prick and intradermal tests
Benzylpenicilloyl polylysine	0.15 mL	2	For prick and intradermal tests
Penicillin dilution 5000 units/mL	0.15 mL	2	For prick and intradermal tests
Diphenhydramine ^b^	50 mg IV × 1 PRN itching or severe reaction	1	
Hydrocortisone ^b^	50 mg IV × 1 PRN severe reaction	1	
Epinephrine 1:1000	0.3 mg IM × 1 PRN severe reaction	1	
**Miscellaneous Component**			
Sterile scratch test devices (e.g., Duo-tip^®^)		5	For prick test
Alcohol swabs		~3–4	To prep area prior to prick and intradermal tests
Pen or marker		1	To mark test area
Ruler		1	To measure wheal size
Patient education card/document		1	Provided to patient

^a^ Core components require seven syringes, five fitted with intradermal needles (note: this is one suggested method, other institutions may prepare kits differently). ^b^ Alternatively, oral options may be given. Diphenhydramine 25–50 mg PO; Prednisone 20–60 mg PO. PO: by mouth; PRN: as needed.
